# Lignosulfonic Acid Exhibits Broadly Anti-HIV-1 Activity – Potential as a Microbicide Candidate for the Prevention of HIV-1 Sexual Transmission

**DOI:** 10.1371/journal.pone.0035906

**Published:** 2012-04-27

**Authors:** Min Qiu, Qin Wang, Ying Chu, Zhongping Yuan, Hongyong Song, Zhiwei Chen, Zhiwei Wu

**Affiliations:** 1 Center for Public Health Research, School of Medicine, Nanjing University, Nanjing, People's Republic of China; 2 State Key Lab of Analytical Chemistry for Life Science, Nanjing University, Nanjing, People's Republic of China; 3 AIDS Institute, Li Ka Shing Faculty of Medicine, The University of Hong Kong, Pokfulam Hong Kong SAR, People's Republic of China; Institut Pasteur of Shanghai, Chinese Academy of Sciences, China

## Abstract

Some secondary metabolites from plants show to have potent inhibitory activities against microbial pathogens, such as human immunodeficiency virus (HIV), herpes simplex virus (HSV), *Treponema pallidum*, *Neisseria gonorrhoeae*, etc. Here we report that lignosulfonic acid (LSA), a polymeric lignin derivative, exhibits potent and broad activity against HIV-1 isolates of diverse subtypes including two North America strains and a number of Chinese clinical isolates values ranging from 21.4 to 633 nM. Distinct from other polyanions, LSA functions as an entry inhibitor with multiple targets on viral gp120 as well as on host receptor CD4 and co-receptors CCR5/CXCR4. LSA blocks viral entry as determined by time-of-drug addiction and cell-cell fusion assays. Moreover, LSA inhibits CD4-gp120 interaction by blocking the binding of antibodies specific for CD4-binding sites (CD4bs) and for the V3 loop of gp120. Similarly, LSA interacts with CCR5 and CXCR4 via its inhibition of specific anti-CCR5 and anti-CXCR4 antibodies, respectively. Interestingly, the combination of LSA with AZT and Nevirapine exhibits synergism in viral inhibition. For the purpose of microbicide development, LSA displays low *in vitro* cytotoxicity to human genital tract epithelial cells, does not stimulate NF-κB activation and has no significant up-regulation of IL-1α/β and IL-8 as compared with N-9. Lastly, LSA shows no adverse effect on the epithelial integrity and the junctional protein expression. Taken together, our findings suggest that LSA can be a potential candidate for tropical microbicide.

## Introduction

Since the advent of AIDS pandemic, efforts have been directed to the search of viral inhibitory molecules to either block virus at the entry portal or disrupt viral life cycle after the entry. Tremendous progress has been made in drugs that inhibit virus through disrupting reverse transcription, integration or proteolytic processing of viral proteins, which witness the introduction of nucleoside analog reverse transcriptase inhibitors (NRTIs), non-nucleoside analog reverse transcriptase inhibitors (NNRTIs), integrase inhibitors and protease inhibitors as Highly Active Antiretroviral Therapy (HAART). The introduction of HAART can effectively keep the viral replication at an undetectable level, thus prolong the life expectancy of the infected and reduce the viral transmission. Comparatively, fewer agents that inhibit viral entry have made to the market.

The infection of HIV-1 is initiated by the viral envelop interaction specifically with its cellular receptor CD4, which leads to further interaction with viral co-receptor CCR5 or CXCR4 [1], [2]. The binding processes are coordinated by HIV-1 envelop conformational changes that are essential for the virus-cell fusion to proceed to productive infection of the host cells [1], [2]. Evidence also suggested that the nonspecific interactions of viral particles and cell surface molecules, such as the heparan sulfate moiety of proteoglycans and cell surface adherent molecules, also play important roles in viral attachment and entry [3]. It is well established that sulfated polyanions (SPs) are potent inhibitors of HIV infection by either competing with cell surface molecules for virus binding or directly interacting with cell surface molecules that are required for the virion attachment or entry [4]. These negatively charged molecules can bind HIV-1 envelop glycoproteins, and cell surface molecules, such as CD4 on the lymphocytes, through charge-charge interactions and thus disrupt viral binding or fusion process [5]–[7]. Heparan and its chemical derivatives were found to inhibit HIV and HSV infection, through binding to viral proteins and probably disrupting the attachment and entry processes [8], [9]. In the case of HSV-1, the heparan sulfate binding to viral gD protein is rather specifically mediated by 3-O-sulfated GlcNp residue that is essential for HSV-1 to penetrate host cells [10]. Another extensively investigated SP is dextran sulfate [8] that potently inhibited HIV-1 replication in cultured CD4^+^ lymphoblastoid cell lines [4]. Mechanistic studies showed that dextran sulfate may act on both virions [11] and target cells [5]. The V3 loop has been reported to be a major HIV-1 region directly interacting with SPs [5], [11]–[15], though other sequences located in the V2, CD4 binding site (CD4bs) and C-terminus of gp120 were also described [16]. SPs' binding to cell-associated molecules were also reported and considered to play roles in viral inhibition as well [5]–[7], [17], [18].

Many of the reported SPs have a linear polysaccharide backbone with varying degrees of sulfation. Current study reported an HIV-1 inhibiting macromolecule that constitutes three-dimensional scaffold polymers composed of sulfated phenylpropanoid monomers. Macromolecular lignin sulfonate presents a multitude of polydispersity that can interact with biomolecules through hydrophobic, hydrogen-bonding, and anionic interactions as other sulfated polyanions (dextran sulfate, heparan sulfate, etc.). Our evidence showed that the polymers may exert the HIV inhibitory activity through multiple bindings with both viral and cell surface molecules and present as potential HIV inhibitors on viral attachment or entry.

## Results

### LSA inhibition of HIV-1 infection *in vitro* and synergism with AZT and nevirapine

The antiviral activity of LSA was tested against two laboratory-adapted HIV-1 strains and a number of clinical isolates on Ghost (3) X4/Hi5 cells using an Env-pseudotyped infection assay. LSA was shown to inhibit both JR-FL (R5-type) and HXB2 (X4-type) at an EC_50_ of 6.323 μg/ml and 1.411 μg/ml, respectively (Table 1). LSA also exhibited inhibitory activities against a panel of diverse clinical isolates derived from infected Chinese patients [19], with EC_50_ values ranging from 0.171 μg/ml to 5.060 μg/ml (Table 1). For all the isolates tested, the EC_50_ values, ranged from 0.171 μg/ml to 6.323 μg/ml, were well below the CC_50_ cytotoxicity values determined in the corresponding cells (Table 2). In contrast, LSA did not have significant inhibitory activity against VSV-G pseudovirus. LA, a LSA precursor, showed less inhibitory activity against all strains (data not shown), demonstrating that sulfonic groups are critical for the antiviral activity.

**Table 1:** LSA inhibition of HIV-1 pseudotyped virus infection of Ghost (3) X4/Hi5 cells.

Virus	Tropism	subtype	EC_50_ (μg/ml)	EC_90_ (μg/ml)
JR-FL	CCR5-tropic	B	6.323	17.047
HXB2	CXCR4-tropic	B	1.411	3.700
CNE6	CCR5-tropic	B'	0.717	2.764
CNE30	CCR5-tropic	B'C	5.055	8.280
CNE50	dual tropic	B'C	0.171	13.455
CNE55	CCR5-tropic	CRF01-AE	2.710	8.019
VSVG pseudovirus	–	–	39.701	>90

**Table 2:** CC_50_ values of cytotoxicity of LSA in human cell lines.

Cell line	CC_50_ value (μg/ml)
MT-2 (human T-cell leukemia cells)	308.64
C33-A (human cervical carcinoma cells)	420.78
Caco-2 (human epithelial colorectal adenocarcinoma cells)	1147.23
Ghost (3) X4/Hi5 (derived from HOS cells)	726.34
VK2/E6E7 (human vaginal epithelial cells)	690.45

We also investigated the inhibitory activities of LSA in combination with RT inhibitors and analyzed their synergistic effects. As shown in Table 3, the combination of LSA with AZT (an NRTI) or nevirapine (an NNRTI) exhibited moderate synergism against JR-FL and synergism against HXB2, respectively.

**Table 3:** CIs for LSA with AZT or nevirapine in Ghost (3) X4/Hi5 cells infected with JR-FL and HXB2 strains.

Combination	Weight Ratio	CI^a^ at 50% inhibition of HIV-1	
		JR-FL	HXB2
LSA/AZT	7666.7:1	0.836	0.445
LSA/nevirapine	230:1	0.743	0.609

a. CI <0.1: very strong synergism; 0.1–0.3: strong synergism; 0.3–0.7: synergism; 0.7–0.85: moderate synergism; 0.85–0.90: slight synergism; 0.9-1.1: Nearly additive and >1.1: antagonism.

### The inhibitory mechanism of LSA

**Figure 1:**
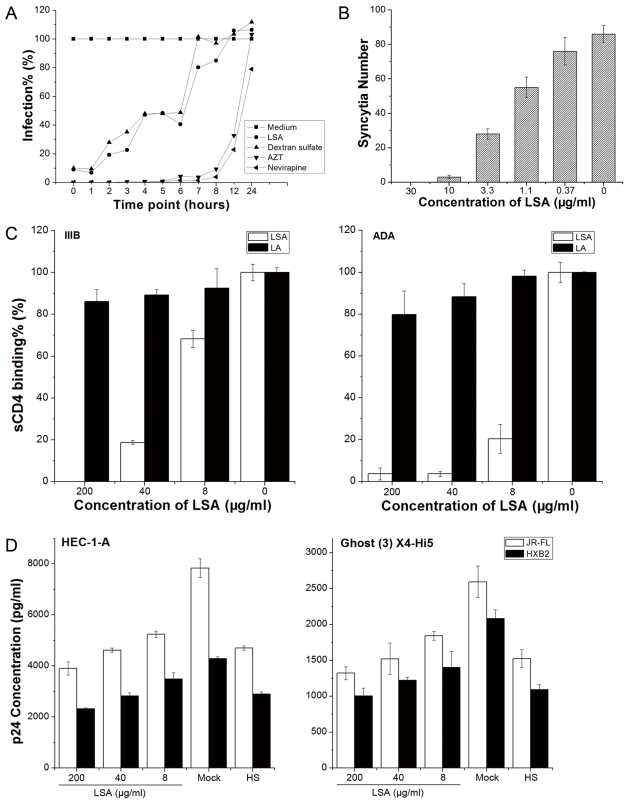
LSA inhibited HIV-1 infection at viral entry stage. A. Time-of-drug-addition assay indicated that LSA blocked HIV-1 infection at an entry stage. B. MT-2-CHO-WT Cell-cell fusion was inhibited in the presence of LSA. C. LSA, not LA blocked sCD4 binding to rgp120s from ADA and IIIB strains. D. LSA inhibited JR-FL and HXB2 HIV-1 virion attachment to Ghost (3) X4/Hi5 and HEC-1-A cells. Heparan sulfate (100 μg/ml) was used as a control. Data shown were the average of three independent experiments. Error bars denoted standard errors of the mean values.

The time-of-drug-addition assay was performed to investigate the inhibitory mechanism of LSA. For comparison, two RT inhibitors, AZT and nevirapine, and an entry inhibitor, dextran sulfate, were used. As shown in Figure 1A, LSA exhibited the similar inhibitory profile as dextran sulfate, but distinct from both RT inhibitors. Viral infection was completely blocked when LSA was added at 1 hour postinfection but significant infection was detected when LSA was added in 2 hour postinfection. As expected, both AZT and nevirapine completely suppressed viral infection when added until 8 hours postinfection. These results demonstrated that LSA inhibited virus at an early stage during binding and entry. We also investigated the activity of LSA in inhibiting gp120-mediated membrane fusion. As shown in Figure 1B, LSA inhibited cell-cell fusion between gp160 expressing CHO-WT cells and MT-2 cells in a dose-dependent manner.

We also determined that LSA could block sCD4 binding to immobilized gp120s (shown in Figure 1C) while LA failed to inhibit at the same molar concentration. However, our data showed differential inhibitory activities against gp120ADA and gp120IIIB primarily in that LSA appeared more efficient in blocking gp120ADA than gp120IIIB from binding to sCD4.

Whether LSA inhibited HIV-1 virion attachment to cells was also studied. The results showed that LSA inhibition of HIV-1 virion attachment was minimal, even at the highest concentration of 200 μg/ml in either CD4-positive Ghost cells or CD4-negative cervical cells, as shown in Figure 1D. Heparan sulfate at 100 μg/ml (HS), serving as a control, exhibited only 30%–50% inhibition against JR-FL and HXB2, consistent with previous report [15].

### LSA inhibited antibody binding to the V3 loop and CD4 binding site on gp120

**Figure 2:**
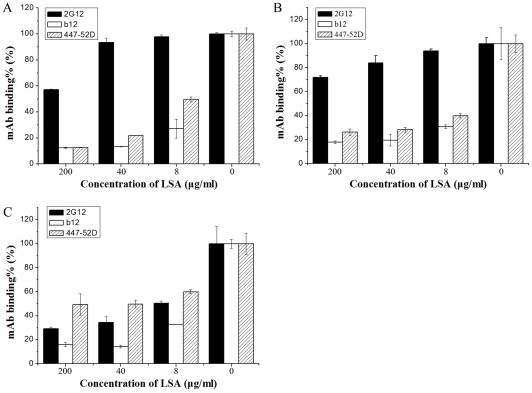
The effect of LSA on the gp120-specific mAb binding. gp120-specific mAbs were incubated in the presence of various concentrations of LSA with immobilized gp120_IIIB_ (A), gp120_ADA_ (B) or gp120_YU-2_ (C) in a 96-well plate. AP-conjugated anti-human secondary antibody was added after excess LSA and the first antibodies were removed. The results were presented as the percentage of mAb binding. Each datum point was the means of triplicate and the experiment was repeated 3 times. A typical experiment was shown. Error bars denote standard error of the mean values.

Studies have demonstrated that the V3 loop on HIV-1 Env is a major region interacting with sulfated polysaccharides via charge-charge interaction [5], [11], [12], [14], [15], [20], though other regions of gp120 may also involve [16]. LSA was investigated for its effect on a panel of neutralizing mAb binding to gp120 by ELISA. V3 specific mAb 447–52D binding to gp120s was significantly reduced by LSA in a dose-dependent manner. Similarly, 447–52D binding to synthetic linear V3 peptides derived from JR-FL and IIIB was also blocked (data not shown). 2G12, a broadly neutralizing mAb specific for mannose carbohydrates, was not significantly inhibited by LSA in its binding to gp120_ADA_ and gp120_IIIB_ but significantly inhibited in binding to gp120_YU2_. The effective inhibition of 2G12 binding to gp120_YU-2_ by LSA probably attributes to the fact that gp120YU-2 lacks a critical glycan required for efficient 2G12 binding causing reduced 2G12 binding affinity [21], which allows more efficient LSA blocking. CD4bs mAb b12 was also inhibited by LSA (Figure 2).

### LSA interacted with cellular CD4 and ECL2 of CCR5/CXCR4

**Figure 3:**
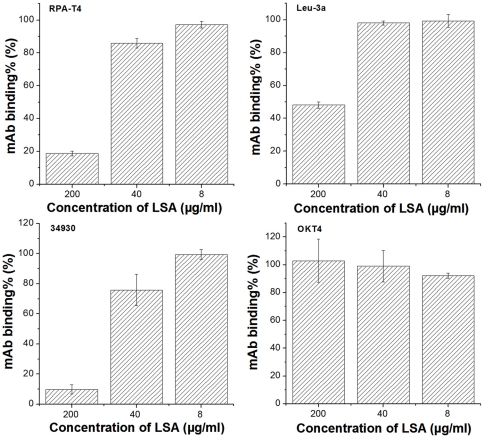
The effect of LSA on anti-CD4 mAb binding. MT-2 cells were incubated with a panel of CD4-specific mAbs with or without the presence of various concentrations of LSA for 30 minutes at 4°C. The bindings of the mAbs to the cell surface CD4 were measured by FACS and expressed as the percentages of mAb binding in the absence of LSA. Error bars represented standard deviations. Each datum point was the means of the triplicate and the experiment was performed 2–3 times. A representative experiment was shown.

We also investigated whether LSA interacted with cell surface CD4 and viral co-receptors. The bindings of four anti-CD4 mAbs (RPA-T4, Leu-3a, 34930 and OKT4) to CD4-expressing MT-2 cells were analyzed in the presence or absence of LSA by FACS. Leu-3a, RPA-T4 and 34930 are HIV-1 neutralizing antibodies, recognizing distinct epitopes. Leu-3a recognizes D1 domain of CD4 at the site overlapping the CDR2 region and RPA-T4 binds the CDR1 and CDR3 while the epitope for 34930 is yet to be defined. OKT4 recognizes an epitope on D3 domain of CD4 and does not block HIV-1 entry. A dose-dependent reduction of the binding of RPA-T4 and 34930 was observed in the presence of LSA as shown in Figure 3. Both RPA-T4 and 34930 binding to MT-2 cells was inhibited by more than 80% at 200 μg/ml LSA while Leu-3a was inhibited by 50% only at the highest concentration of 200 ug/ml. In contrast, LSA showed no effect on OKT4 binding.

**Figure 4:**
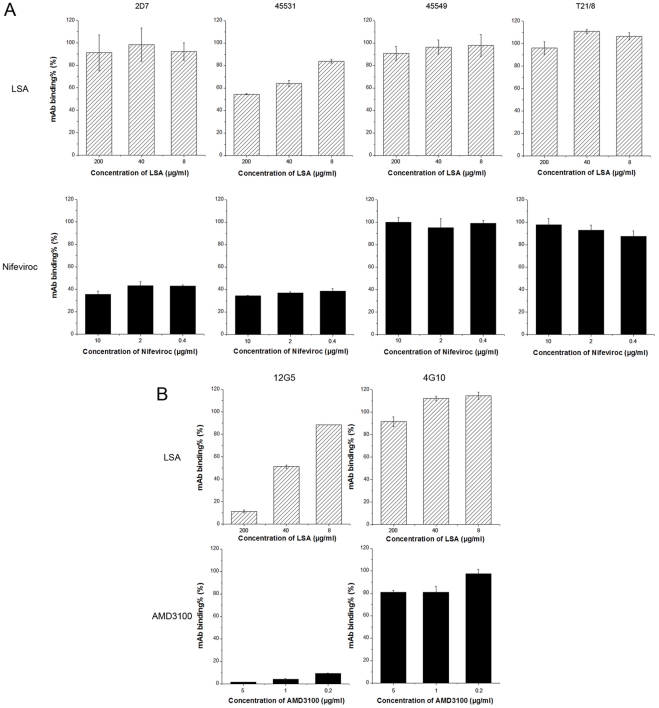
The effect of LSA on the anti-CCR5/CXCR4 mAb binding. CCR5-specific mAb binding to Ghost (3) X4/Hi5 cells (A) were inhibited by LSA (dashed block) or Nifeviroc, a CCR5 antagonist serving as a control (solid block), or CXCR4-specific mAb bindings to Ghost (3) X4/Hi5 cells (B) were inhibited by LSA (dashed block) or AMD3100, a CXCR4 antagonist serving as a control (solid block). Ghost (3) X4/Hi5 cells in suspension were incubated with anti-CCR5/CXCR4 mAbs in the presence of various concentrations of LSA or Nifeviroc for 30 minutes at 4°C. The binding of the mAbs to the cell surface co-receptors was measured by FACS. Data shown were the average of three independent experiments. Error bars denote standard errors of individuals.

mAbs specific for the N-terminals and extracellular loop 2 (ECL2) of the co-receptors were chosen to investigate whether their bindings to Ghost (3) X4/Hi5 cells were inhibited by LSA since these two regions are most important for HIV-1 gp120 binding and viral entry [22]. Firstly, we examined the inhibitory effect of LSA on the binding of a number of HIV-1 inhibitory mAbs to CCR5 (Figure 4A). mAbs 2D7 and 45531 recognize the N-terminal of ECL2 (ECL2A) and the C-terminal of ECL2 (ECL2B), respectively, while mAb T21/8 recognizes the N-terminal of CCR5. mAb 45549 is reactive to multi-domains of CCR5. LSA slightly reduced only mAb 45531 binding to CCR5 (46.4% inhibition) at the highest concentration and had no effect on other mAbs. Nifeviroc, a CCR5 antagonist, used as a positive control, showed strong inhibitory activity on 2D7 and 45531 binding.

LSA significantly blocked 12G5 binding to CXCR4 while showed no significant effect on 4G10, as shown in Figure 4B. 12G5 recognizes an epitope overlapping ECL2 of CXCR4 and 4G10 is specific for the N-terminal of CXCR4. AMD3100, a CXCR4 antagonist, was used as the positive control and exhibited potent inhibition of 12G5 binding.

### LSA was characterized by low cytotoxicity *in vitro*

The *in vitro* cytotoxicity of LSA was analyzed on human vaginal and cervical epithelial cell lines (VK2/E6E7 and C33-A), Caco-2, human T-cell leukemia cells (MT-2) and indicator cells (Ghost (3) X4/Hi5), respectively. For all 5 cell lines tested, LSA showed low cytotoxicity with CC_50_ values ranging from 308 to 1147 μg/ml (Table 2), presenting appreciable Therapeutic Indexes as a potential antiviral drug.

**Figure 5:**
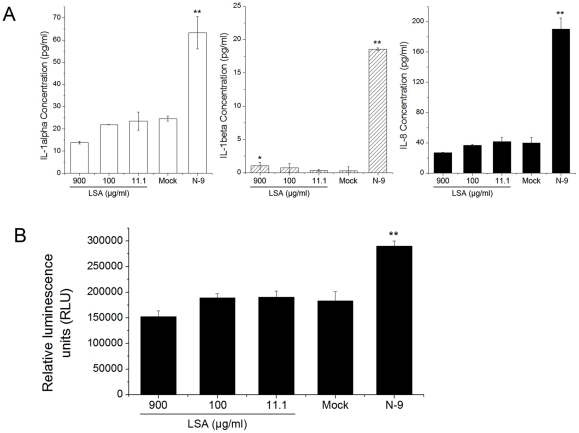
LSA effect on NF-κB activation and modulation of IL-8 production. A. IL-1α/β and IL-8 secretions by VK2/E6E7 cells after exposure to LSA for 6 hours. B. Quantification of NF-κB activation in VK2/E6E7 cells post-incubation with various concentration of LSA using a NF-κB luciferase reporter plasmid. N-9 (10 μg/ml) was used as a positive control for the stimulation of NF-κB, cytokines and chemokines productions. *: P<0.05 and **: P<0.01.

LSA also did not elevate IL-1α and IL-8 expression above its basal level, as shown in Figure 5A, although slightly up-regulate IL-1β, which might be caused by LSA acute cytotoxicity. N-9, a surfactant and the first microbicide candidate, served as the positive control and showed significant up-regulation of IL-1α/β and IL-8 in VK2/E6E7 cells. Overall, LSA did not alter cytokines/chemokines profile of genital epithelial cells significantly in comparison with N-9.

The effect of LSA on NF-κB activation in genital tract epithelial cells was also investigated. We did not find that NF-κB activity in VK2/E6E7 cells was augmented after 24 hours LSA treatment, in contrast to the N-9 treatment that NF-κB activated luciferase expression was significantly elevated (Figure 5B).

### LSA did not cause the destruction of epithelium integrity

**Figure 6:**
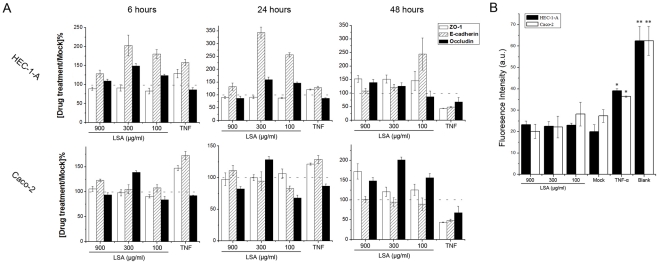
LSA did not destroy epithelium integration. A. ZO-1, E-cadherin and occludin expressions in HEC-1-A and Caco-2 cells after LSA treatment for 6, 24 and 48 hours. B. Lucifer Yellow leakage through HEC-1-A or Caco-2 monolayers after treatment with serial concentrations of PSM for 48 hours. TNF-α treatment (100 ng/ml) was used as a positive control. Each datum point was mean of triplicate and the experiment was repeated twice. A representative experiment was shown. *: P<0.05 and **: P<0.01.

For topical antiviral application, low toxicity and none disruptive to mucosal epithelium of the antiviral agent are considered critical. Therefore, we investigated whether LSA would affect tight junction protein expression important for the integrity of epithelium using HEC-1-A and Caco-2 cells. HEC-1-A and Caco-2 cells were treated with serial concentrations of LSA or 100 ng/ml TNF-α (as a positive control) for 6, 24 and 48 hours, and target protein expressions were analyzed by qRT-PCR. 6 hour-treatment did not affect tight junction protein expression, as shown in Figure 6A. After 24 hours exposure to LSA, ZO-1 and occluding expressions were not affected significantly in both cells, while E-cadherin was markedly up-regulated in HEC-1-A cells. Similarly, the genes for the three proteins were not down-regulated significantly in the presence of LSA in comparison with TNF-α which down-regulated the expressions of all three proteins after 48 hours treatment.

We also investigated the effects of LSA on the epithelial permeability by evaluating Lucifer Yellow leakage in HEC-1-A and Caco-2 cells. As shown in Figure 6B, LSA did not increase Lucifer Yellow leakage in HEC-1-A and Caco-2 cell monolayers compared with the mock wells which were not exposed to the drugs. Cell monolayers which were incubated with 100 ng/ml TNF-α were more permissive for leakage of Lucifer Yellow, consistent with a previous report [23]. In conclusion, LSA did not compromise the integrity of epithelial cell monolayers at non-cytotoxic concentration.

## Discussion

Lignin is a polymeric natural product derived from wood, and an integral part of the secondary cell walls of all terrestrial plants. Unlike heparan sulfate, a linear copolymer of glucosamine (GlcNp) and glucuronic acid (GlcAp) linked in a1–4 manner. Sulfated lignin is a three-dimensional scaffold polymer composed of substituted phenylpropanoid monomers. Macromolecular lignin sulfate presents a multitude of polydispersity that can interact with biomolecules through hydrophobic, hydrogen-bonding, and anionic interactions. In this report, we presented studies on inhibitory activities of the sulfated lignin against various HIV-1 isolates, its mechanisms of action and *in vitro* cytotoxicity.

Six HIV-1 isolates (JR-FL, HXB2, CNE6, CNE30, CNE50, and CNE55) representing clades B, B', B'C and AE recombinant viral subtypes were chosen for the inhibitory study. JR-FL, a CCR5-tropic virus, and HXB2, an CXCR4-tropic virus, were well-characterized isolates derived from North American subtype B while all CNE viruses were derived from circulating isolates from Chinese patients [19]. LSA exhibited potent inhibitory activities against all six HIV-1 isolates (Table 1). In contrast to dextran sulfate [24], we did not found great differential inhibitory activities against X4 and R5 viruses, suggesting that the V3 charge is not the only determinant for LSA activity, which is consistent with the fact that LSA binds multiple sites on gp120. In addition, LSA, in combination with AZT or nevirapine, exhibited synergism or modest synergism against HIV-1_JR-FL_ and HIV-1_HXB2_ strains, which broaden PSM potential application as an antiviral agent in combination with other drugs.

Time-of-drug-addition study suggests that LSA exerted its inhibitory activity at an early stage of viral entry with a window time of about 2 hours and that the LSA inhibitory profile overlapped with that of dextran sulfate. The LSA inhibition of HIV-mediated syncytia formation further demonstrated that LSA is an entry inhibitor, consistent with current understanding on the inhibitory mechanisms of sulfated polysaccharides [15], [25]–[27].

The V3 domain has been shown previously to be a major Env determinant interacting with sulfated polysaccharides through charge-mediated interaction [11]. Our study showed that the binding of 447-52D, a neutralizing anti-V3 mAb was inhibited by LSA in a dose-dependent manner, suggesting that the positively charged LSA is likely interacting with the negatively charged V3 loop sequence. The lack of LA, a derivative that lacks sulfonic moiety, in inhibitory activity further supports the view that charge-mediated interaction plays important roles. This observation suggested that LSA is distinct from dextran sulfate that was previously shown not to inhibit 447-52D binding to gp120 [28]. The current study also showed that LSA interacted with CD4bs on gp120. CD4bs antibody F105 was almost completely blocked from binding to gp120 by LSA. b12, another CD4bs mAb, was also inhibited by LSA for binding to gp120, consistent with the observation that LSA blocked sCD4 binding to gp120s. LSA effectively blocks 12G5 binding to CXCR4 but only slightly inhibited mAb 45531 binding to CCR5. It is unlikely that blocking of 12G5 binding was caused by LSA binding to the antibody since 4G10, recognizing the N-terminus of CXCR4, was not affected by LSA. Moulard *et al.* [28] reported that dextran sulfate also blocked 12G5 binding to CXCR4 and found that CXCR4 virus was significantly more sensitive to dextran sulfate inhibition *in vitro*. However, we did not observe consistently differential inhibition of X4 and R5 virus infections by LSA. Together, the analysis of LSA inhibition of mab binding to gp120, CD4 and coreceptors suggests that LSA inhibition is likely mediated by binding to multiple targets, consistent with its three dimensional scaffold structure.

However, we observed that LSA only slightly inhibited virion attachment to both CD4-positive and -negative cells. Monder *et al*. [29] observed that HIV-1 attachment was minimally influenced by the presence of cellular CD4 and suggested that nonspecific cellular adhesion molecules may play important roles in mediating initial virus-cell interaction to bring virions to the CD4 proximity to form high affinity binding. Our data are consistent with Monder's observation. Several studies have reported that SPs inhibited HIV-1 infection by disrupting the interaction between gp120 and CD4, by either binding to CD4 [5], [7] or to gp120 [16], [28]. Lynch *et al*. [6] reported that dextran sulfate and other SPs prevented HIV-1 infection by disrupting the interaction between gp120 and CD4 molecules and suggested that the SP binding to CD4 is the predominant mechanism of inhibition. Lynch *et al*. [6] failed to detect SP-rgp120 binding but reported weak interaction between SP and a V3 peptide. Crublet *et al*. [16] mapped four gp120 domains that interacted with heparan sulfate, V2, V3, a bridging sheet induced by CD4 binding and a sequence at the C-terminus of gp120. Harrop *et al*. [30] reported that heparan sulfate interacted with viral gp120 rather than the receptor-CD4.

**Figure 7:**
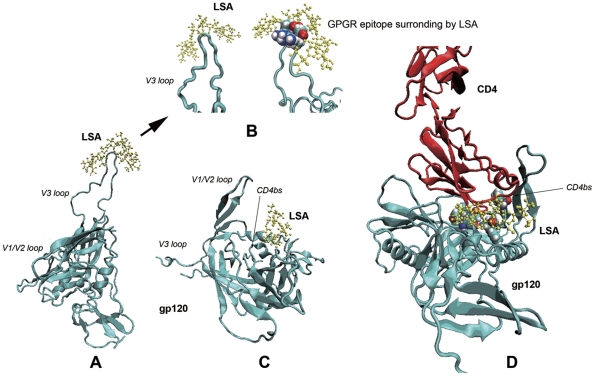
Molecular modelling of two optimal docking predictions. A. Docking prediction of LSA surrounding gp120 V3 loop. B. Details of the interaction between V3 loop and LSA molecule (four amino acids G-P-G-R displayed in CPK mode interacting with LSA molecule). C. Docking prediction showing LSA binding to the proximity of CD4 binding site (CDbs) (the CD4bs residues, Glu370, Ile371, Trp427, Val430 and Gly473, were labeled by CPK mode). D. LSA might block or interfere CD4 binding to gp120. Red, cyan and yellow represented CD4 molecule, gp120 and LSA molecule, respectively.

To facilitate our understanding on how LSA might interact with its target molecules and exert its viral inhibitory activity, we generated a computer-aided three dimensional structure of LSA and docked the structure with the complete structures of both gp120_JR-FL_ and gp120_HXB2_ that were reconstructed by homology modeling using published data [31], as shown in Figure 7. The docking analysis suggested that the most stable and optimized docking was LSA in complex with the tip of V3 loop, which predicted that LSA would create a steric clash for the interacting V3 mAbs, such as 447–52D. Other possible binding sites were predicted to be in the vicinity of CD4bs. Even though LSA did not insert into the pocket of CD4bs, this binding manner might interfere with the CD4 and anti-CD4bs mAbs binding to this area. The computational prediction was consistent with experimental data. The selective inhibitions of mAb bindings to gp120 ruled out the possibility that the LSA inhibitions were in fact mediated by LSA binding to mAbs instead of gp120. This conclusion would also apply to LSA inhibition of mAb binding to CD4 and the co-receptors CCR5 or CXCR4.

Prophylactic use of therapeutic viral reverse transcriptase inhibitor has been successfully tested in clinical trials and demonstrated to be safe and effective in preventing sexual transmission of HIV-1 [32]. Combination of anti-viral agents with distinct working mechanisms is considered to provide even better protection. The synergistic effects of LSA in combination with a number of RT inhibitors, together with low toxicity to epithelial cells, make LSA an interesting candidate as a topical microbicide. Topical application will avoid the shortfalls, such as poor ability in penetrating blood barriers and toxicity to lymphocytes [33]–[36], associated with many SPs [37]. Our observations that LSA did not disrupt the epithelial monolayer integrity and decrease the tight junction protein expression and modulate inflammatory cytokine expression significantly further support this notion. In addition, our preliminary data showed that LSA did not disturb the growth of lactobacillus, an important component of vaginal microflora (data not shown). Together, our observations suggest that LSA deserve further investigation as a tropical microbicide candidate.

## Methods

### Reagents, cell lines and plasmids

Lignosulfonic acid (LSA), lignin alkali (LA), heparan sulfate, DEAE-dextran, dextran sulfate, azidothymidine (AZT), AMD3100, gelatin, p-nitrophenyl phosphate (p-NPP), N-9 were purchased from Sigma-Aldrich (St. Louis, USA). Lipofectamine 2000, TRIzol, Lucifer Yellow were from Invitrogen (Carlsbad, USA). Nevirapine was obtained from National Institutes for Food and Drug Control in China (Beijing, China). Nifeviroc was a kind gift from Shanghai Target Drug Ltd. (Shanghai, China). Cell counting kit-8 (CCK-8) was purchased from Dojindo Laboratories (Kumamoto, Japan). Glo Lysis buffer and luciferase assay kit were obtained from Promega Bio-technology (Madison, USA). Recombinant gp120s (rgp120s) and soluble CD4 (sCD4) and were purchased from Immunodiagnostics, Inc. (Woburn, USA). The HIV p24 ELISA kit was purchased from Shanghai Qifa Biotech Ltd. (Shanghai, China). gp120-specific monoclonal antibodies (mAbs) b12, 2G12, 447-52D and F105 were from AIDS Research and Reference Reagent Program, National Institute of Health (NIH, USA). Anti-CD4 mAb RPA-T4, anti-CCR5 mAb 2D7 and anti-CXCR4 12G5 were purchased from BD Biosciences (San Jose, USA). Human rTNF-α, anti-CD4 mAb 34930, anti-CCR5 mAbs 45531 and 45549 and normal human IgG control were from R&D Systems (Minneapolis, USA). Anti-CD4 mAb OKT4 and PE-labelled Leu-3a, goat-anti-human IgG FITC, goat-anti-mouse IgG FITC, purified mouse IgG1/IgG2a/IgG2b isotype control and IL-1α/β and IL-8 ELISA kits were obtained from eBioscience (San Diego, USA). Anti-CCR5 mAb T21/8 and anti-CXCR4 mAb 4G10 were purchased from Santa Cruz Biotechnology (Santa Cruz, USA). Alkaline phosphatase labeled goat-anti-human and goat-anti-mouse IgG was purchased from Zymed (South San Francisco, USA).

HEC-293T, VK2/E6E7, Caco-2 and C33-A cells were purchased from American Type Culture Collection (ATCC). Ghost (3) X4/Hi5, MT-2 and CHO-WT cells were obtained from AIDS Research and Reference Reagent Program, NIH. Plasmid pNL4-3 E^-^R^-^ Luc and HIV-1/VSV-G Env were kindly provided by Dr. Linqi Zhang at Tsinghua University, China. NF-κB-luc reporter plasmid was purchased from Clotech (Palo Alto, USA).

### Antiviral activity assay

The Env-pseudotyped viruses were produced by transient co-transfection of HEC-293T cells with pNL4-3 E^-^R^-^ Luc and Env-encoding plasmids as reported [24], [38], and the 50% tissue culture infective dose (TCID_50_) of infectious pseudovirions was determined as reported previously [39], [40]. Compounds against pseudotyped HIV-1 virus were determined as described [40]. Briefly, cultural medium containing serially diluted LSA was mixed with 200 TCID_50_ pseudovirus in 96-well plates and incubated for 30 min. All drug dilutions were in triplicate. 10^4^ cells in 100 μl medium containing DEAE-dextran (final concentration 5 μg/ml) were dispensed to each well and the plates were incubated for 48 hours. The level of viral infection was quantified by measurement of relative luminescence units (RLU) using luciferase assay kit by GloMax-96 Microplate Luminometer (Promega, Madison, USA). The half maximal effective concentration (EC_50_) were calculated using CalcuSyn software [41].

### Synergy analysis

AZT, nevirapine and LSA were tested individually in series concentrations in Ghost (3) Hi5/X4 cells and the EC_50_ values of the single drugs were calculated. Two drugs (AZT/LSA and nevirapine/LSA) combinations were tested at a fixed weight concentration ratio, which was optimized to give the greatest synergism over a range of serial dilutions. The EC_50_ values of single drugs and the combination index (CI) of the two drugs were calculated using CalcuSyn software [41] according to the method of Chou-Talalay [42]. The synergy was estimated by CI values [42].

### *in vitro* cytotoxicity assay

The *in vitro* cytotoxicity of LSA was measured on various cell lines using CCK-8 kit in accordance with the manufacturer's instruction. Briefly, 10^4^ cells/well were seeded into 96-well plates and cultured overnight. Drugs in serial concentrations were added in triplicate, and the cell culture was kept for 48 hours. 10 μl CCK-8 working solution was then added into each well and the plates were incubated for 4 hours and OD values were measured at 450 nm using TECAN Infinite M200 microplate reader (Männedorf, Switzerland). The 50% cytotoxicity concentrations (CC_50_) were calculated using CalcuSyn [41].

### Time-of-drug-addition assay

A time-of-drug-addition assay was performed as described elsewhere [43] to investigate the mechanism of the LSA antiviral activity when the drug was added at various time points after virus infection. Briefly, 10^4^ Ghost (3) X4/Hi5 cells cultured overnight were incubated with 200 TCID_50_ JR-FL viruses in 96-well plates. Test compounds or culture medium alone (negative control) were added at different time points post viral infection. Dextran sulfate was used at 100 μg/ml, nevirapine at 2 μg/ml, AZT at 0.5 μg/ml, and LSA at 40 μg/ml. The RLUs were measured as described above.

### Inhibition of cell-cell fusion

Inhibition of fusion of MT-2 cells (CD4^+^ CXCR4^+^) and CHO-WT cells stably transfected with HIV-1 gp160 was determined using a syncytium formation assay [44] with modifications. Briefly, 5×10^5^ gp160-expressing CHO-WT cells were mixed and incubated with an equal number of MT-2 cells in the presence or absence of LSA in serial concentrations for 24 hours. The monolayers were fixed with 10% formaldehyde-PBS for 3 minutes and stained with Giemsa dye. The syncytia were counted under inverted microscope.

### Inhibition of sCD4 binding to rgp120s

The effects of LSA on sCD4 binding to gp120 were analyzed by solid phase enzyme-linked immunosorbent assay (ELISA). 8 μg/ml gp120 in 50 μl 0.1 M NaHCO_3_ (pH 9.6) was coated to 96-well plate at 4°C overnight. The plate was then blocked with 3% nonfat dry milk in TS buffer (0.14 M NaCl, 0.01 M Tris, pH 7.0) at 37°C for 1 hour. LSA diluted in TS buffer was added in duplicate and incubated for 30 minutes, followed by the addition of 100 ng sCD4 and further incubation at 37°C for 1 hour. After washing five times with TS buffer, bound sCD4 was detected by anti-CD4 OKT4 mAbs (2 μg/ml) followed by AP-conjugated goat-anti-mouse IgG (2 μg/ml). Fifty microliters of p-NPP solution were added into each well and the absorbance at 405 nm was measured by a microplate reader.

### Interaction of LSA with CD4, CCR5 and CXCR4

To determine the interaction between LSA and CCR5/CXCR4, Ghost (3) X4/Hi5 cells were digested with 0.5 mM EDTA and 10^6^ cells were incubated with 2 μg mAbs specific for CD4, CCR5 or CXCR4 in the presence or absence of LSA at 4°C for 30 minutes. The cells were washed twice with PBS containing 2% FBS and then stained with 2 μg goat-anti-mouse FITC-conjugated mAb at 4°C for 30 minutes. For nonspecific control, cells were stained in parallel with isotype control mAbs. The cells were then fixed with 2% paraformaldehyde solution and analyzed with a FACSCalibur (BD, San Jose, USA). The percentage of mAb binding was calculated based on the mean fluorescence intensity (MFI).

### Interaction of LSA with viral gp120

The interaction between LSA and rgp120s was investigated by ELISA. Briefly, 96-well plates were coated with 50 ng/well gp120_IIIB_ or gp120_ADA_ in 0.1 M Tris buffer (pH 8.8) at 4°C overnight. The plates were washed with TS buffer five times and blocked with 1mg/ml BSA and gelatin (0.1 mg/ml) in TS buffer at 37°C for 1 hour. Serial concentrations of LSA were added and incubated, followed by the addition of 10 ng/well mAbs and secondary antibody as described earlier. Color development was carried out as described before.

### Virion attachment assay

LSA inhibition of virion attachment was performed as previously described [45], but modified. 5×10^4^ HEC-1-A or Ghost (3) X4/Hi5 cells/well were seeded into a 24-well plate and cultured overnight. The cell monolayers were then exposed to 50 ng p24 virus solution and incubated in the presence or absence of LSA at 37°C for 1 hour. Addition of 100 μg/ml heparan sulfate was set as positive control. The cell monolayer was washed five times and then lysed immediately using 0.5 ml cell lysis buffer (1% NP-40, 100 μg/ml BSA in PBS). The lysate was centrifuged and p24 level in the supernatant was determined using p24 core antigen ELISA kit.

### RNA extraction and real-time quantitative reverse transcription polymerase chain reaction (qRT-PCR)

Total RNA was extracted using TRIzol reagent. cDNA was reverse-transcribed in a 20 μl volume using RT-PCR kit (TOYOBO, Osaka, Japan). Real-time PCR was performed in triplicate on ABI Prism 7300 Sequence Detection System using the SYBR Green PCR Master Mix (TOYOBO, Osaka, Japan) according to the manufacturer's protocol. Human ZO-1, E-cadherin and Claudin mRNA was standardized against housekeeping gene GAPDH. qPCR Primers were from RTPrimerDB [46] and listed as follows: ZO-1 Forward AAGTCACACTGGTGAAATCC, ZO-1 Reverse CTCTTGCTGCCAAACTATCT; E-cadherin Forward AGGCCAAGCAGCAGTACATT, E-cadherin Reverse ATTCACATCCAGCACATCCA; Occludin Fw CATTGCCATCTTTGCCTGTG, Occludin Reverse AGCCATAACCATAGCCATAGC; GAPDH Forward TGCACCACCAACTGCTTAGC, GAPDH Reverse GGCATGGACTGTGGTCATGAG.

### Lucifer yellow leakage assay

Epithelial cell monolayers were prepared in 0.4 μm-pore Millicell hanging cell culture inserts (Millipore Corporation, Bedford, USA) in 24-well plates (Corning, New York, USA) as described [47]. Various LSA doses and 100 ng/ml TNF-α were pre-incubated in Millicell apical chambers for 48 hours, followed by two PBS washings of the apical and basolateral chambers. 200 μl 50μg/ml Lucifer Yellow solution, prepared in PBS, was added to the apical chambers and 1300 μl PBS to the basolateral ones. Non-cell-layer-inserts were set as mock controls. The plates were incubated at 37°C, 5%CO_2_ for 3 hours. Lucifer yellow leakage into basolateral chambers were measured using Hitachi F7000 spectrofluorometer (Tokyo, Japan) with excitation wavelength of 428 nm and emission wavelength of 536 nm. All treatments were in duplicate.

### Quantification of NF-κB activation and cytokines and chemokines expression

Quantification of NF-κB activation in cells treated with LSA and N-9 was detected by using NF-κB-luc reportor plasmid. The plasmid was transiently transfected into VK2/E6E7 cells using Lipofectamine 2000 transfection reagent. After 24 hours, LSA at various concentrations and 10 μg/ml N-9 were added, and the cells were cultured for another 24 hours. The level of NF-κB activation was quantified by measurement of relative luminescence units (RLU) as described above.

To determine IL-1α/β and IL-8 expression, VK2/E6E7 cells were seeded into 24-well plates and treated with various concentrations of LSA and 10 μg/ml N-9 as positive control. After 6 hours incubation, the supernatants were harvested and centrifuged to remove cellular debris and IL-1α/β and IL-8 concentrations were measured via ELISA. Their concentrations were calculated by quadratic regression analysis based on logarithmically transformed optical densities.

### Structural modeling and docking analysis

The molecular models of LSA and LA were generated and energy-optimized using ChemOffice Ultra 8.03 software. The structures of JR-FL (R5 type) and HXB2 (X4 type) gp120s containing V3 loops were reconstructed using Modeller 9v8 program [48] according to the template (PDB: 2B4C) and sequence information from HIV Database (http://www.hiv.lanl.gov/content/index). The charge density distribution of the molecules was analyzed by Discovery Studio v2.1 software.

The molecular docking simulation of LSA onto the gp120 was performed by Autodock 4.0 software [49]. A force-field-based empirical free energy scoring function was used in this program. The Lamarckian Genetic Algorithm (LGA) was used as a search engine. The active site was defined using AutoGrid program. Both ligands and the target protein were kept rigid. The resultant LSA-gp120 conformations were ranked and categorized based on the Intermolecular energy criteria. VMD 1.8.7 software was using to view and prepare the figures [50].

### Statistical analysis

Statistical analysis was performed using a two-tailed *t* test. The significance level was set at *P* = 0.05.
